# Activation of PPARα by Oral Clofibrate Increases Renal Fatty Acid Oxidation in Developing Pigs

**DOI:** 10.3390/ijms18122663

**Published:** 2017-12-08

**Authors:** Yonghui He, Imad Khan, Xiumei Bai, Jack Odle, Lin Xi

**Affiliations:** Laboratory of Developmental Nutrition, Department of Animal Sciences, North Carolina State University, Raleigh, NC 27695, USA; hyonghui@163.com (Y.H.); ikhan@ncsu.edu (I.K.); bxm8302@126.com (X.B.); jodle@ncsu.edu (J.O.)

**Keywords:** peroxisome proliferator-activated receptor α (PPARα), clofibrate, fatty acid β-oxidation, pigs

## Abstract

The objective of this study was to evaluate the effects of peroxisome proliferator-activated receptor α (PPARα) activation by clofibrate on both mitochondrial and peroxisomal fatty acid oxidation in the developing kidney. Ten newborn pigs from 5 litters were randomly assigned to two groups and fed either 5 mL of a control vehicle (2% Tween 80) or a vehicle containing clofibrate (75 mg/kg body weight, treatment). The pigs received oral gavage daily for three days. In vitro fatty acid oxidation was then measured in kidneys with and without mitochondria inhibitors (antimycin A and rotenone) using [1-^14^C]-labeled oleic acid (C18:1) and erucic acid (C22:1) as substrates. Clofibrate significantly stimulated C18:1 and C22:1 oxidation in mitochondria (*p* < 0.001) but not in peroxisomes. In addition, the oxidation rate of C18:1 was greater in mitochondria than peroxisomes, while the oxidation of C22:1 was higher in peroxisomes than mitochondria (*p* < 0.001). Consistent with the increase in fatty acid oxidation, the mRNA abundance and enzyme activity of carnitine palmitoyltransferase I (CPT I) in mitochondria were increased. Although mRNA of mitochondrial 3-hydroxy-3-methylglutaryl-coenzyme A synthase (mHMGCS) was increased, the β-hydroxybutyrate concentration measured in kidneys did not increase in pigs treated with clofibrate. These findings indicate that PPARα activation stimulates renal fatty acid oxidation but not ketogenesis.

## 1. Introduction

The kidney is an organ with a high energy requirement due to its central role in the elimination of water-soluble metabolic waste products. Thus, energy metabolism is very active and important for renal physiology. In support of the high energy metabolism, renal fatty acid oxidation and carnitine biosynthesis are very active, generating ketone bodies when fatty acids are catabolized and in maintaining carnitine homeostasis, respectively [1]. Recently, a strong link between impaired renal energy metabolism and chronic kidney disease has been highly identified [2,3].

Peroxisome proliferator-activated receptor α (PPARα), a member of a large nuclear receptor superfamily, is expressed primarily in the liver, the intestine, and the kidney [4,5]. The critical role of PPARα activation in regulation of hepatic fatty acid oxidation, lipid metabolism, and inflammatory and vascular responses has been well studied [6]. In contrast with the liver, however, the data on the role of PPARα activation in the regulation of renal fatty acid oxidation and metabolism is scant, especially for developing animals. By comparison, both mitochondrial and peroxisomal β-oxidation enzymes are expressed in the liver and the kidney, but the enzymes in peroxisomes are less abundant in the kidney than in the liver. The response of mitochondrial and peroxisomal β-oxidation enzymes to PPARα activation in the kidney is also moderate [7]. Despite all this, the importance of peroxisomal β-oxidation in short-, long-, and very long-chain fatty acids has been well recognized. Moreover, the essential role of PPARα-induction of fatty acid metabolism in the prevention of renal ischemia and renal damage induced by drugs has been observed in rodent species [8,9,10].

Potential ligands for the PPARα transcription factor include fatty acids, eicosanoids, and pharmacological drugs such as the fibrates. Clofibrate is a potent PPARα activator that stimulates peroxisome proliferation and increases fatty acid oxidation in rodent species. The target genes of PPARα encode enzymes involved in peroxisomal and mitochondrial β-oxidation and ketone body synthesis. The peroxisome proliferation elicited by fibrates has drawn much attention because peroxisome proliferation has been associated with oxidative stress and hepatocellular carcinoma [11]. However, less is known about the impacts of the agonist in the kidney. Fatty acids are the preferred energy substrate for the kidney, and defects in fatty acid oxidation and mitochondrial and peroxisomal dysfunction are involved in acute renal injury and chronic disease. Indeed, PPARα signaling may play a protective role in acute free fatty acid-associated renal tubule toxicity [12]. PPARα activation has been recognized as essential for kidney function under both healthy and pathophysiological states [7].

Data regarding inborn errors in the kidney such as neonatal urea cycle defects and disorders of long-chain fatty acid oxidation associated with energy deficiency in infants is very limited in the literature. Understanding the renal kinetics and adaptation of energy metabolism is very important for human infant health. The domestic neonatal pig (*Sus scrofa*) ranks among the most prominent research models for the study of pediatric nutrition and metabolism due to the similarity of human infant and piglet physiology [13]. Unlike rodent species, the peroxisome proliferation and hepatocarcinogenic potencies of clofibrate are not observed in the livers of humans or pigs [14,15]. Peroxisomal β-oxidation (enzymes) increase with the age in the renal cortex of suckling rat pups, and this might be involved in PPARα-mediated mechanisms [16]. Similarly, previous work from our laboratory showed that fatty acid β-oxidation capacity was increased with age in the kidney of pigs as well, and the capacity was higher during the preweaning period than in adults [17]. The enzymatic responses to PPARα activation also were compared in the heart, kidney, and liver of pigs in our previous work, but effects of the activation on fatty acid oxidative metabolism were not determined. Promoting energy supply and thermogenesis after birth are critical for the survivor of neonatal piglets [17]. Therefore, to provide basic knowledge on the regulation of energy metabolism in the developing kidney, the present study assessed changes in peroxisomal and mitochondrial long-chain fatty acid oxidation in the kidney during early development in response to the activation of PPAR by clofibrate.

## 2. Results

### 2.1. β-Hydroxybutyrate Concentration

**Figure 1:**
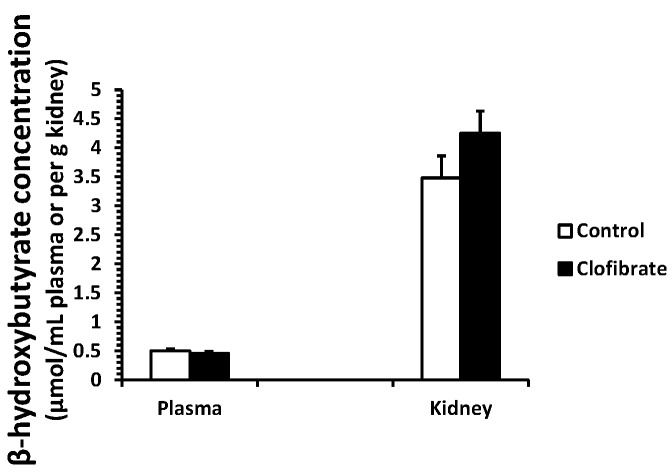
β-Hydroxybutyrate concentration in plasma and kidneys of neonatal piglets. Values are means ± SE (*n* = 5).

No differences were detected in β-hydroxybutyrate concentration measured in plasma and kidney tissues between control and clofibrate-treated pigs (*p* > 0.05). The concentration of β-hydroxybutyrate was on average 8-fold higher in kidney tissue compared with plasma (Figure 1).

### 2.2. Fatty Acid Oxidation In Vitro

**Figure 2:**
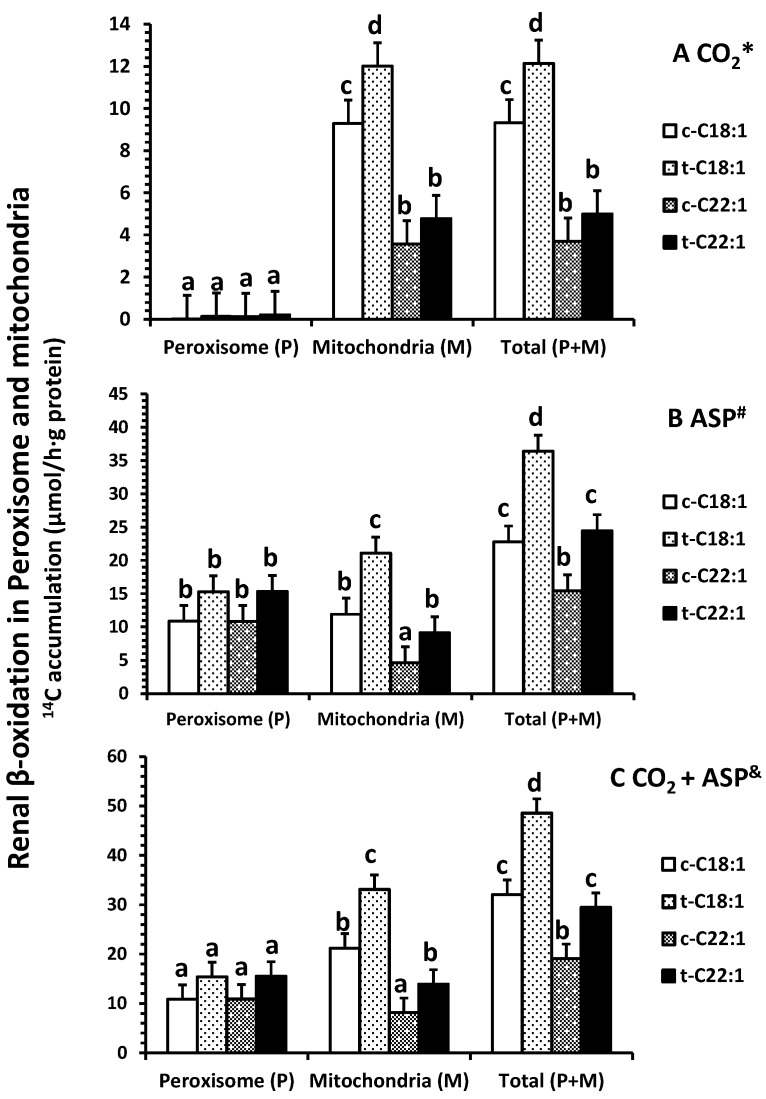
Effects of oral clofibrate on renal β-oxidation (^14^C accumulation in CO_2_ * (**A**); ASP ^#^ (**B**) and CO_2_ + ASP ^&^ (**C**)) in peroxisomes and mitochondria of neonatal pigs. Values are least square means ± SEM (*n* = 5). Abbreviations: ASP: acid soluble product; c-C18:1: control with oleate; t-C18:1: treatment with oleate; c-C22:1: control with erucate; t-C22:1: treatment with erucate. * Clofibrate effect (*p* < 0.037) and fatty acid effect (*p* < 0.0001); ^#^ Clofibrate effect (*p* < 0.0001) and fatty acid effect (*p* < 0.0001); ^&^ Clofibrate effect (*p* < 0.0001) and fatty acid effect (*p* < 0.0001). Column with different letters differ (*p* < 0.05).

Clofibrate had no effects on the accumulation of ^14^CO_2_ in peroxisomes from either [1-^14^C]-C18:1 or C22:1 oxidation (*p* > 0.05), but the accumulation in mitochondria and in homogenates (a total of peroxisomes and mitochondria) from [1-^14^C]-C18:1 was significantly higher in clofibrate-treated than control piglets (*p* < 0.05; Figure 2A). The ^14^CO_2_ accumulation from [1-^14^C]-C18:1 and C22:1 oxidation were on average 133- and 25-fold higher in mitochondria than peroxisomes (*p* < 0.0025). In addition, the ^14^CO_2_ accumulation in mitochondria or homogenates were 2.5-fold greater from [1-^14^C]-C18:1 than C22:1 (*p* < 0.0009).

Clofibrate tended to increase the accumulation of ^14^C in acid-soluble product (ASP) in peroxisomes from both [1-^14^C]-C18:1 and C22:1 oxidation (*p* = 0.06), but the accumulation of ^14^C in ASP from C18:1 and C22:1 in mitochondria and in homogenate were increased in clofibrate-treated compared to the control pigs (*p* < 0.006; Figure 2B). There was no difference between C18:1 and C22:1 in ^14^C-ASP accumulation in peroxisomes, but the ^14^C-ASP accumulation from C18:1 was greater than that from C22:1 in mitochondria. The accumulations of ^14^C-ASP in the homogenates also were 1.5-fold higher from [1-^14^C]-C18:1 compared with C22:1 (*p* < 0.001).

By combining both ^14^CO_2_ and ^14^C-ASP (Figure 2C), the total oxidation (CO_2_ + ASP) from either C18:1 or C22:1 was not affected by clofibrate in peroxisomes (*p* > 0.05). However, clofibrate increased mitochondrial oxidation of C18:1 by 56% and C22:1 by 70%. Thus, the total oxidation in homogenates was significantly higher from clofibrate-treated than control piglets (*p* < 0.001). The oxidation from C18:1 was on average 1.7-fold greater than that from C22:1 (*p* < 0.03).

**Figure 3:**
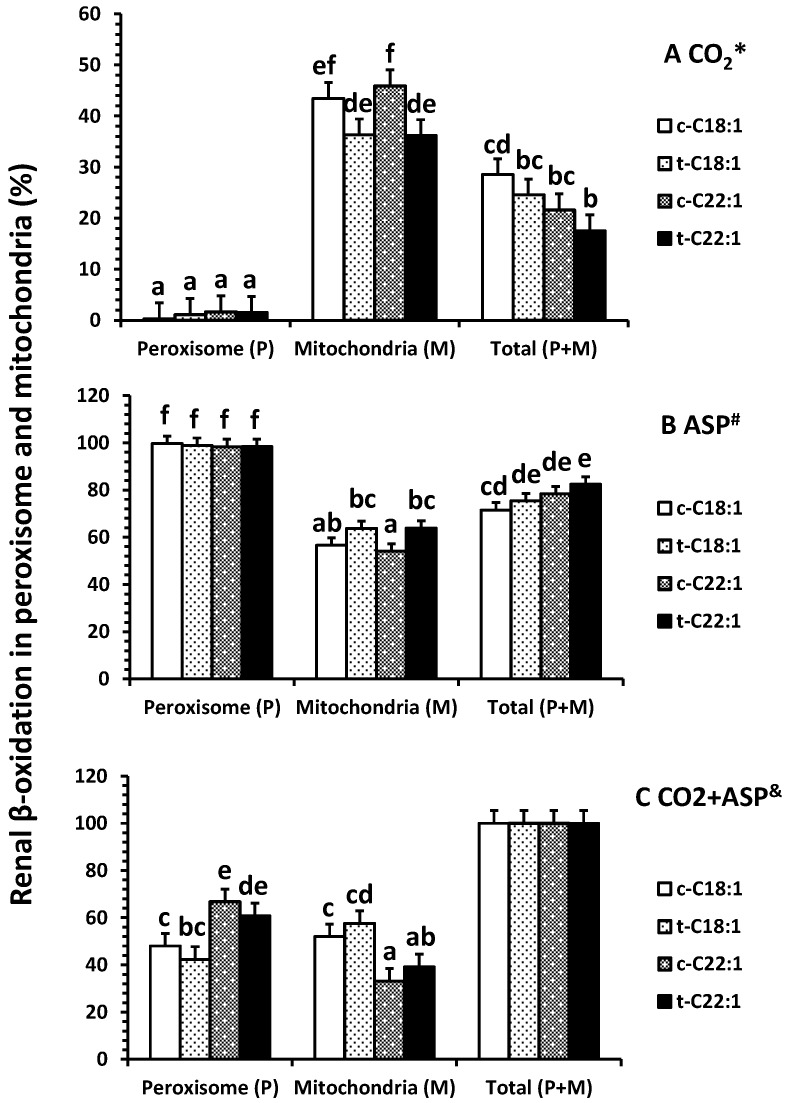
Percentage distribution of renal β-oxidation (% of ^14^C accumulation in CO_2_ * (**A**), ASP ^#^ (**B**), and CO_2_ + ASP ^&^ (**C**)) in peroxisomes and mitochondria of neonatal pigs. Values are least square means ± SEM (*n* = 5). Abbreviations: ASP: acid soluble product; c-C18:1: control with oleate; t-C18:1: treatment with oleate; c-C22:1: control with erucate; t-C22:1: treatment with erucate. * Clofibrate effect (*p* < 0.040) and fatty acid effect (*p* = 0.39); ^#^ Clofibrate effect (*p* < 0.0001) and fatty acid effect (*p* = 0.39); ^&^ Clofibrate effect (*p* = 1.0) and fatty acid effect (*p* = 1.0). Column with different letters differ (*p* < 0.05).

No difference was observed in the percentage of ^14^C accumulation in CO_2_ (less than 2%) in peroxisomes (*p* = 0.9), but clofibrate reduced the percentage of accumulation of C22:1 in CO_2_ in mitochondria (*p* < 0.01) (Figure 3A). Over 98% of the oxidative metabolites were ASP in peroxisomes, while only about 60% (54–64%) of the ASP was detected in mitochondria (Figure 3B). Clofibrate administration did not affect the percentage of ASP from C18:1 (*p* = 0.13) but increased the ASP from C22:1 significantly (*p* < 0.04) (Figure 3B). The percentage of total oxidation (CO_2_ + ASP) from C22:1 in peroxisomes was 1.5-fold higher than that from C18:1, and the percentage of total oxidation from C18:1 in mitochondria was 1.5-fold higher than that from C22:1 (Figure 3C).

### 2.3. Renal Enzyme Activity

**Figure 4:**
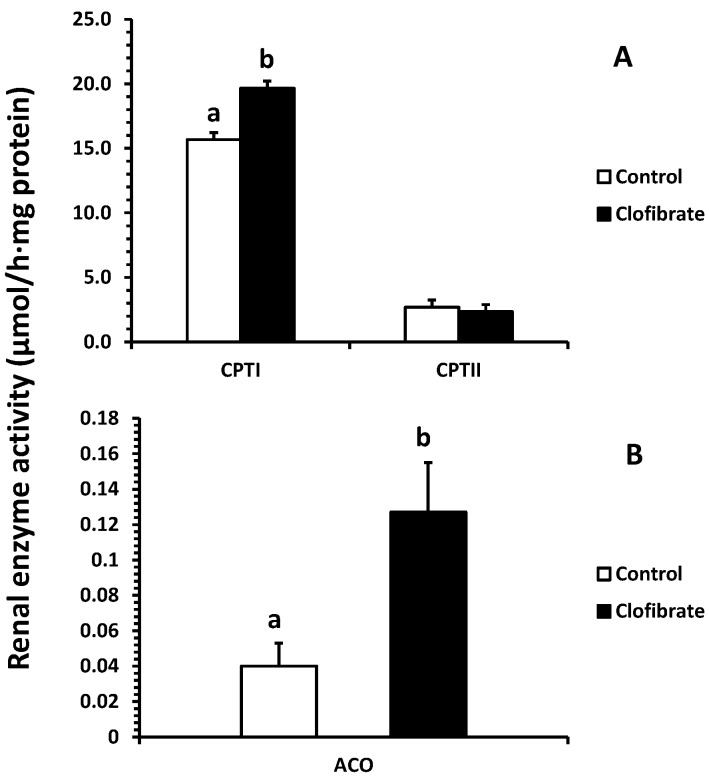
Effects of oral clofibrate on renal enzyme activity in neonatal pigs. Values are least square means ± SEM (*n* = 5). CPT I and CPT II, carnitine palmitoyltransferase I and II (**A**), and ACO, acyl-CoA oxidase (**B**). Columns with different letters are different (*p* < 0.05).

The activity of carnitine palmitoyltransferase I (CPT I) was increased 25% by clofibrate (*p* < 0.05), but no effect on the activity of CPT II was detected (*p* > 0.05; Figure 4A). The activity of acyl-CoA oxidase (ACO) was increased 2.2-fold in clofibrate-treated pigs (*p* < 0.05; Figure 4B).

### 2.4. Renal mRNA Enrichment

**Figure 5:**
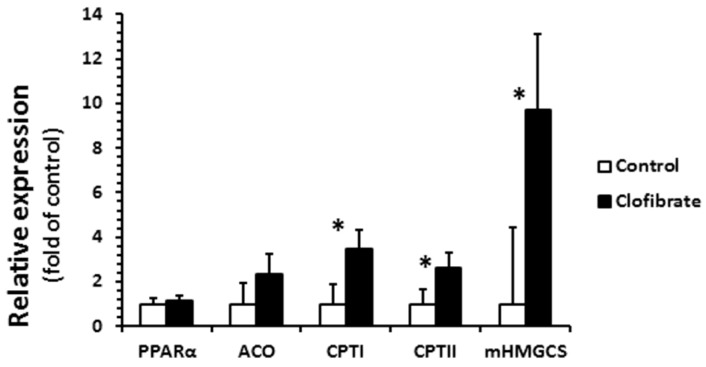
Renal mRNA abundance in pigs with and without oral clofibrate. Values are least square means ± SEM (*n* = 5). PPARα: peroxisome proliferator-activated receptor α; ACO: acyl-CoA oxidase; CPT I and CPT II: carnitine palmitoyltransferase I and II; mHMGCS: mitochondrial 3-hydroxy-3-methylglutaryl-coenzyme A synthase. * Significant difference between control and treatment groups (*p* < 0.05).

Clofibrate administration had a great impact on the relative mRNA abundance of CPT I, CPT II, and mHMG-CoA, but had no effects on PPARα and ACO (Figure 5). The mRNA enrichments of CPT I, CPTII, and mitochondrial 3-hydroxy-3-methylglutaryl-CoA synthase (mHMG-CoA) were 3.5-, 2.6-, and 9.7-fold greater from clofibrate-treated pigs than control pigs (*p* < 0.001).

## 3. Discussion

Activation of PPARα by oral clofibrate administration to newborn piglets resulted in a significant increase in renal fatty acid β-oxidation. Similar observations were reported in humans and rats [18]. Fatty acid β-oxidation is the primary pathway of ATP production for the kidney to meet its daily function requirement. Therefore, this result implied that PPARα could play an important regulatory role in ATP production and energy metabolism in the developing kidney. We also noticed that the induction profiles were different in mitochondria and peroxisomes for the long- and very long-chain fatty acids, suggesting that the response of renal fatty acid β-oxidation to PPARα activation depends on the subcellular and substrates.

The activation had no significant impact on the fatty acid β-oxidation (^14^C accumulation in CO_2_ or/and ASP) in renal peroxisomes, although the ACO activity increased 2.2-fold in clofibrate-treated piglets. Only a tendency of increase in ASP (*p* = 0.06) was observed, and the mild response of peroxisomal β-oxidation to the PPARα agonist was similar to that reported in adult rats [19]. As in mitochondria, fatty acid β-oxidation in peroxisomes involves multiple enzymes that ultimately yield acetyl-CoA [20]. However, the peroxisomal fatty acid β-oxidation is not coupled with ATP synthesis and catalase is required for H_2_O_2_ produced in peroxisomes by transferring electrons to O_2_. It was reported that the activation of PPARα had no influence on catalase activity in 14-day-old piglets [21], and catalase increases fast after birth [22]. This result could be related to the catalase or other enzymes in β-oxidation system of peroxisomes such as the bifunctional protein and 3-ketoacyl-CoA thiolase during development. In addition, we did not find any difference in renal PPARα and ACO mRNA enrichments between control and clofibrate-treated piglets. The low response of PPARα and ACO mRNA to clofibrate induction was observed in the livers of newborn, 24-hour-old, and 4-day-old fasted neonatal piglets [21,23,24]. Besides, the ACO activity measured in kidneys of 14-day-old control pigs was not different from pigs treated with clofibrate [21]. Because the rates of mitochondrial and peroxisomal β-oxidation of palmitate change during postnatal development and food deprivation in pig kidneys [22], age or physiological status and even species could contribute to these differences.

A similar ^14^C-accumulation rate in CO_2_ or/and ASP from both C18:1 and C22:1 was detected in peroxisomes, indicating that the chain-length of these two fatty acids had no effects on peroxismal fatty acid β-oxidation. However, the percentage of peroxisomal fatty acid β-oxidation increased with the increase in the fatty acid chain-length. The percentage of β-oxidation of C22:1 was on average 40% higher than that of C18:1, although the total fatty acid oxidation rate had no difference. A similar result was detected in the liver [23], demonstrating that C22:1 has a preference to be oxidized in peroxisomes. The preference for C22:1 appeared to be associated with the affinity of fatty acid activation systems for long-chain fatty acid and very-long-chain fatty acid identified in rat [25]. It was very interesting that a high percentage (about 42–67%) of the fatty acids were oxidized in renal peroxisomes with 98–99% as ASP and 1–2% as CO_2_, and the activation of PPARα had no influence on the percentage distribution of fatty acid oxidation. The contribution of peroxisomal fatty acid β-oxidation to the total fatty acid β-oxidation in the kidney was similar to that measured in the liver (40–47) and 2-fold higher than that in rats (20–35% [26]).

Mitochondrial fatty acid oxidation was increased significantly by the activation of PPARα induced by clofibrate administration. Consistent with the increase in fatty acid β-oxidation, the CPT I activity was increased by 25% and mRNA expression was increased 3.5-fold. In addition, the chain-length of fatty acid significantly affected mitochondrial β-oxidation, and the ^14^C-accumulations were much greater from C18:1 than C22:1 in both of CO_2_ (2.6-fold) and ASP (2.3-fold). Similar results were observed in livers of PPARα-activated neonatal pigs with clofibrate administration [23]. Swine milk fat is known to be composed of mainly long chain fatty acids (LCFAs) and very long chain fatty acids (VLCFAs). These results indicate that mitochondrial oxidation of LCFAs provides an important source of energy for kidneys, and activation of PPARα could promote the utilization of LCFAs and VLCFAs in developing kidneys.

The ^14^CO_2_ accumulation rates from C18:1 and C22:1 (µmol/h·g protein) were on average 64% and 50% higher in the kidney (10.7 and 4.3) than in the liver (3.9 and 2.2; [23]), while the ^14^C accumulations in ASP from C18:1 and C22:1 were 52% and 55% greater in the liver (44.9 and 30.8; [23]) than in the kidney (29.6 and 19.9). It was recently demonstrated that, in rat kidneys, proximal tubules do not generate energy via glycolysis and are completely dependent on oxidative phosphorylation for ATP production, although energy production is primarily from fuels such as lactate, glutamine, and free fatty acids [27]. On the other hand, fatty acid elongation can occur in both livers and kidneys, but it was reported that the specific activity of the fatty acid elongation in the kidney is about 30% compared to the liver. Different incorporation rates [1-^14^C] acetate into fatty acids were observed in the mitochondria elongation system between livers and kidneys in the presence of nicotinamide adenine dinucleotide + hydrogen (NADH), nicotinamide adenine dinucleotide phosphate + hydrogen (NADPH), or both NADH and NADPH as the hydrogen donor [28]. Thus, the results demonstrated that fatty acid catabolic metabolism in mitochondria and citric acid cycle is the primary emergent source in developing kidneys and that activation of PPARα might have a benefit to kidney development via improving fatty acid utilization. Compared with kidneys, the liver may need to produce more ASP in which acetate was found to be one of the primary product in piglets [29].

The mitochondrial 3-hydroxy-3-methylglutaryl-CoA synthase (mHMGCS) mRNA increased 9.7-fold in clofibrate-treated pigs, but the induction of mHMGCS had no influence on plasma and renal β-hydroxybutyrate concentrations. Although the activity of mHMGCS was not measured in this study, available evidence confirms that the enzyme activity in the liver remains low until the weaned age of pigs [30]. Ketone bodies are transferred in and out of cells by monocarboxylate transporter 1. In wild-type mice, treatment with WY 14,643 increased mRNA concentrations of monocarboxylate transporter 1 in the liver, the small intestine, and the kidney, but no upregulation was observed in PPARα-null mice [31]. This suggested that activation of PPARα could potentially promote ketone body production and transfer from organs to plasma. However, we found that β-hydroxybutyrate concentration was 8-fold higher in the kidney tissue than plasma, suggesting that the contribution of the kidney to plasma ketone bodies is minimal in this species. It has been well known that suckling pigs are hypoketonemic despite elevated dietary fat after birth [30].

## 4. Materials and Methods

### 4.1. Experiment Design and Animal Model

All experimental procedures were approved by the North Carolina State University Animal Care and Use Committee. Ten male newborn pigs (Landrace × Yorkshire × Duroc), 2 from each of 5 L, were used in this experiment. The selected newborn piglets (Body weight (BW) = 1.61 ± 0.06 kg) were allocated randomly into two treatments: control and clofibrate. The control piglets were orogastrically gavaged with 2 mL of 2% Tween 80, and the clofibrate-treated piglets were orogastrically gavaged to 2 mL of 2% Tween 80 containing clofibrate (75 mg/kg BW; Cayman Chemicals, Ann Arbor, MI, USA) at 8:00 a.m. of each day for 4 days as described previously [23]. All piglets were kept with their dams and siblings at the North Carolina State University Swine Educational Unit in Raleigh, North Carolina during the experiment. The piglets were euthanized by AVMA-approved electrocution on Day 4 after gavaging and feeding, and kidney and blood samples were collected. Fresh kidney samples were collected and stored in a homogenate buffer, and extra kidney samples were immersed in liquid nitrogen and stored at −80 °C. The blood was sampled with vacutainer containing sodium heparin and centrifuged at 2500 rpm × 10 min. The plasma was collected and stored at −20 °C.

### 4.2. β-Hydroxybutyrate Concentration

A BioVision β-hydroxybutyrate assay kit (K632-100; BioVision, Milpitas, CA, USA) was used to measure the β-hydroxybutyrate concentration in the plasma and kidney samples. The standard curve and samples were prepared according to the BioVision assay procedure and allowed to develop at room temperature for 30 min. The samples were measured with a BioTek reader (Synergy HT, Winooski, VT, USA) at an absorbance of 450 nm.

### 4.3. Fatty Acid Oxidation In Vitro

Fresh kidney homogenates (~5 mg) were incubated in 3 mL of reverse Krebs–Henseleit bicarbonate medium with or without rotenone and antimycin A (10 + 50 μmol/L), blockers of mitochondrial respiratory system. Mitochondrial and peroxisomal fatty acid oxidations were measured in the medium using either [1-^14^C]-labeled oleic acid (C18:1) or erucic acid (C22:1) purchased from American Radiolabeled Chemicals (ARC; Saint Louis, MO, USA) as substrate. The biochemical and radio-chemical purities of both C18:1 and C22:1 were greater than 99% based on TLC and HPLC analyses. The fatty acids were bound to fatty acid-free BSA (5:1, molar ratio) and dissolved in the reaction medium. The measurements were performed in 25 mL Erlenmeyer flasks containing 2 mL of the reaction medium. The medium was incubated with 2 µmol [1-^14^C]-C18:1 (0.98 kBq/µmol) or [1-^14^C]-C22:1 (1.37 kBq/µmol). The incubation was stopped after 30 min by the addition of 0.5 mL of 35% HClO_4_. The ^14^C accumulation in CO_2_ and acid-soluble products (ASP) were collected, processed, and analyzed by liquid scintillation spectrometry (Beckman LS 6000IC, Fullerton, CA, USA) according to the procedures by Lin et al. [24].

### 4.4. CPTI Activity

Kidney mitochondria were isolated from fresh samples. The samples were homogenized in an isolation buffer and centrifuged with a gradient centrifugation [32]. The mitochondria pellet was collected, and the protein concentration was determined using the biuret method as previously described [32]. The CPTI activity was assayed in the mitochondria at 30 °C with 80 μmol/L palmitoyl-CoA following the method used previously [32]. The assays were performed with or without supplementation of 4.7 μg/mL of malonyl-CoA. The assay was initiated by the addition of 20 μL of ^3^H-carnitine (166.5 kBq/μmol) purchased from ARC and terminated with the addition of 4 mL of 6% HClO_4_ after 6 min incubation. The activity was determined by measuring the ^3^H-labeled palmitoyl-carnitine generated from the reactions. The radioactivity was determined using the Beckman liquid scintillation spectrometry (Beckman LS 6000IC, Fullerton, CA, USA).

### 4.5. ACO Activity

The fatty acyl-CoA oxidase (ACO) activity was measured by using a fluorometric procedure with scopoletin, a fluorescing compound as described previously [24]. The reduction of the ACO produced H_2_O_2_ was coupled to the oxidation of scopoletin to its non-fluorescing product. The control and treatment kidney samples were prepared as described previously [32] and were incubated at 37 °C for 20 min. A standard curve was generated consisting of (0–0.1 μm) concentrations of H_2_O_2_. The samples were measured with a BioTek reader (Synergy HT, Winooski, VT, USA) with an emission at 460 nm and an excitation at 360 nm.

### 4.6. mRNA Expression

Total mRNA was extracted using guanidine isothiocynate and phenol, and was quantified using NanoDrop spectrometer (Thermo Scientific, Wilmington, DE, USA). The mRNA was treated with Turbo DNase (Ambion, Austin, TX, USA) and transcribed using iScripTM Select cDNA synthesis kit (Bio-Rad Laboratories, Hercules, CA, USA). Primers were designed with the use of GenBank as described previously [32]. The mRNA abundances were measured with MyiQ Single Color RT-PCR (Bio-Rad Laboratories, Hercules, CA, USA).

### 4.7. Statistical Analysis

Data from plasma β-hydroxybutyrate, tissue enzyme activity and mRNA enrichment assays, were analyzed using the GLM procedure of SAS (Proprietary Software 9.3 (TS1M1), SAS Institute Inc., Cary, NC, USA) according to a randomized complete block design with 2 treatments (control and clofibrate), blocked by litter. Data from in vitro fatty acid oxidation measurements were analyzed with a split-plot design, including a main plot (control vs. clofibrate) in randomized blocks and a subplot modeling fatty acid chain length (C18:1 vs. C22:1) effects, subcellular (mitochondria vs. peroxisomes) differences, and interactions. Multiple comparisons between treatments were performed using Tukey’s test, with significance declared when *p* ≤ 0.05 and tendencies noted when 0.05 ≤ *p* ≤ 0.1.

## 5. Conclusions

Activation of PPARα by clofibrate resulted in a greater increase in mitochondrial long-chain fatty acid oxidation in developing kidneys. The increase was elicited with induced enzyme activity and mRNA expression implies that PPARα activation could improve renal energy utilization during development. More than 40% of the catabolic metabolism occurred in mitochondria and citric acid cycle, suggesting that mitochondrial fatty acid oxidation plays a primary role in energy generation in developing kidneys. However, the activation did not alter the β-hydroxybutyrate concentration in plasma or kidneys.

## Abbreviations

C18:1	oleic acid
C20:1	erucic acid
PPARα	peroxisome proliferator-activated receptor α
mHMGCS	mitochondrial 3-hydroxy-3-methylglutaryl-coenzyme A synthase
CPT	carnitine palmitoyltransferase
